# Supplementary material for: Posthemorrhagic Hydrocephalus in Patients with Subarachnoid Hemorrhage Occurs Independently of CSF Osmolality
Source: Int J Mol Sci. 2023 Jul 14;24(14):11476. doi: 10.3390/ijms241411476 (PMC10380704; doi:10.3390/ijms241411476)
Supplement: Supplementary file 1 [file ijms-24-11476-s001.zip › ijms-2454026-supplementary.pdf]

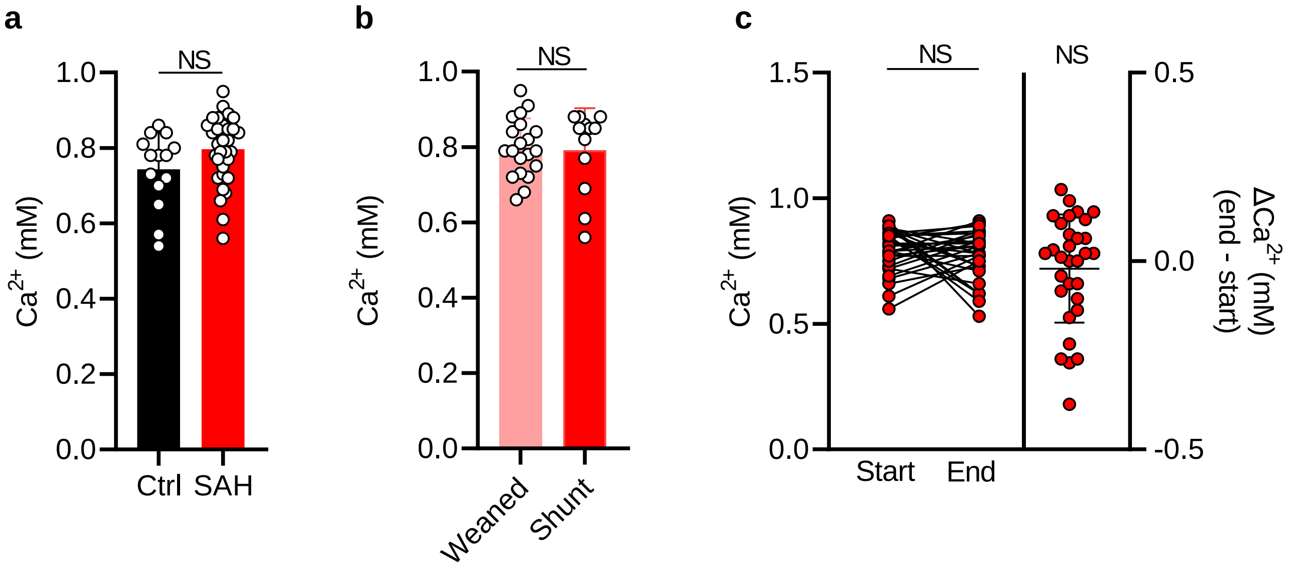

Figure S1. CSF concentration of Ca^{2+}. (**a**) Concentration of Ca^{2+} in CSF from patients with SAH (0.80 ± 0.09 mM, n = 32) and healthy control subjects (0.74 ± 1.00 mM, n = 14, P = 0.08). (**b**) CSF concentration of Ca^{2+} in SAH patients with successful EVD weaning (0.80 ± 0.08 mM, n = 20) and shunt insertion due to chronic PHH development (0.79 ± 0.11 mM, n = 12, P = 0.68). (**c**) CSF concentration of Ca^{2+} across the whole SAH patient group divided into start samples (0.79 ± 0.09 mM, n = 31) and end samples (0.77 ± 0.10 mM, n = 31, P = 0.79, left panel). The average Ca^{2+} change amounted to 0.02 ± 0.14 mM (n = 31, P = 0.89, right panel). Data are presented as mean ± standard deviation. For unpaired CSF samples, statistical significance was tested with an unpaired two-tailed t-test or a Mann-Whitney test, depending on normality. A Wilcoxon matched-pairs signed rank test was employed for paired CSF samples. A one-sample Wilcoxon signed rank test was employed to determine the average Ca^{2+} change. Ctrl; control, SAH; subarachnoid hemorrhage. NS = not significant.

Supplementary Figure S1

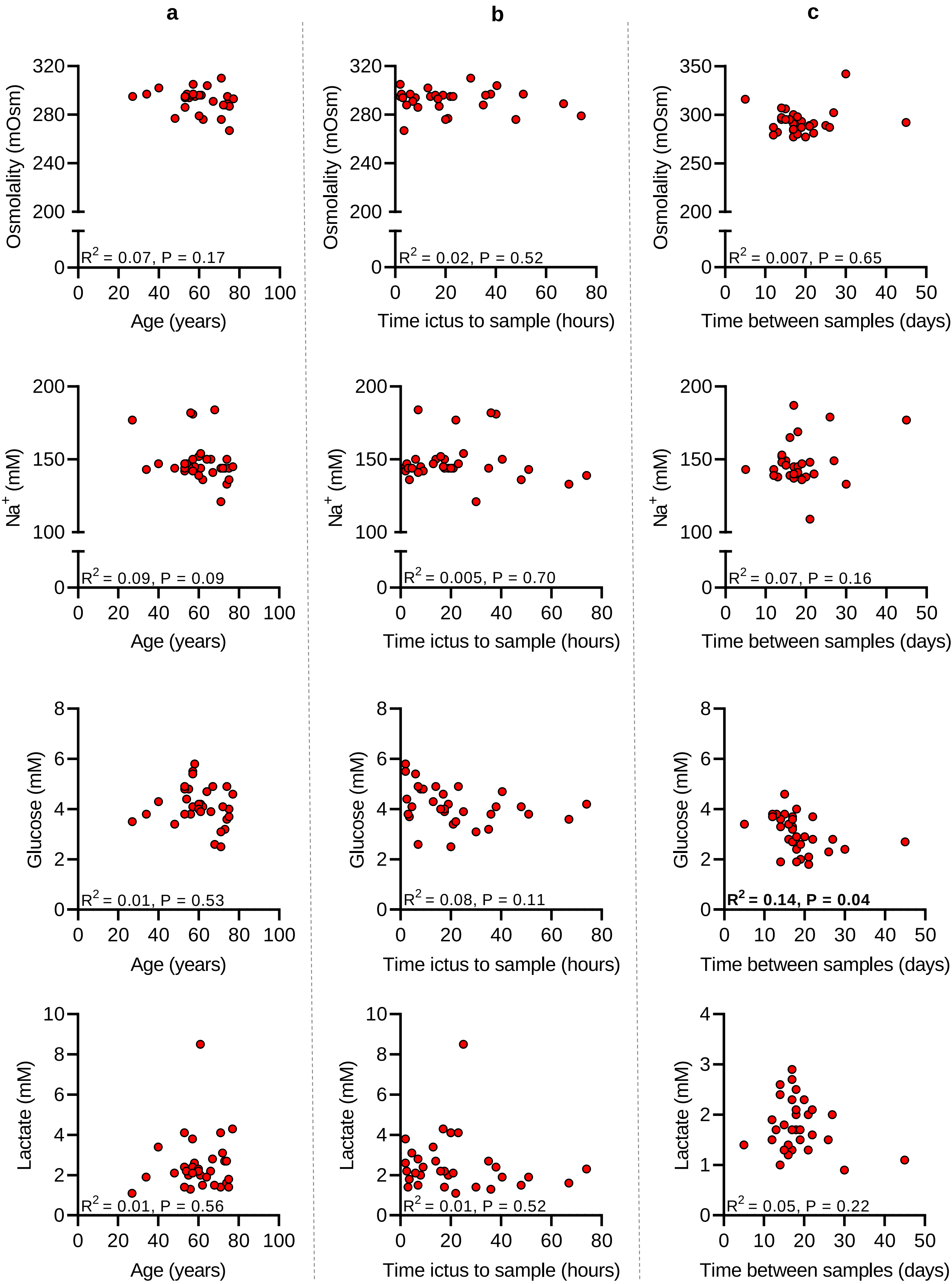

Supplementary Figure S2

Figure S2. Correlation analysis. Correlation between CSF osmolality, Na^+, glucose, and lactate levels with patient age (**a**), time from ictus to first CSF sample (**b**), and time between the first and second CSF sample (**c**). Start samples were employed for correlation analysis with patient age (**a**) and time from ictus to first CSF sample (**b**), while end samples were employed for correlation analysis of time between the first and second CSF sample (**c**). All SAH patients were included in the analysis (n = 29-32), statistical evaluation with Pearson correlation coefficient. Significant P values are highlighted in bold.

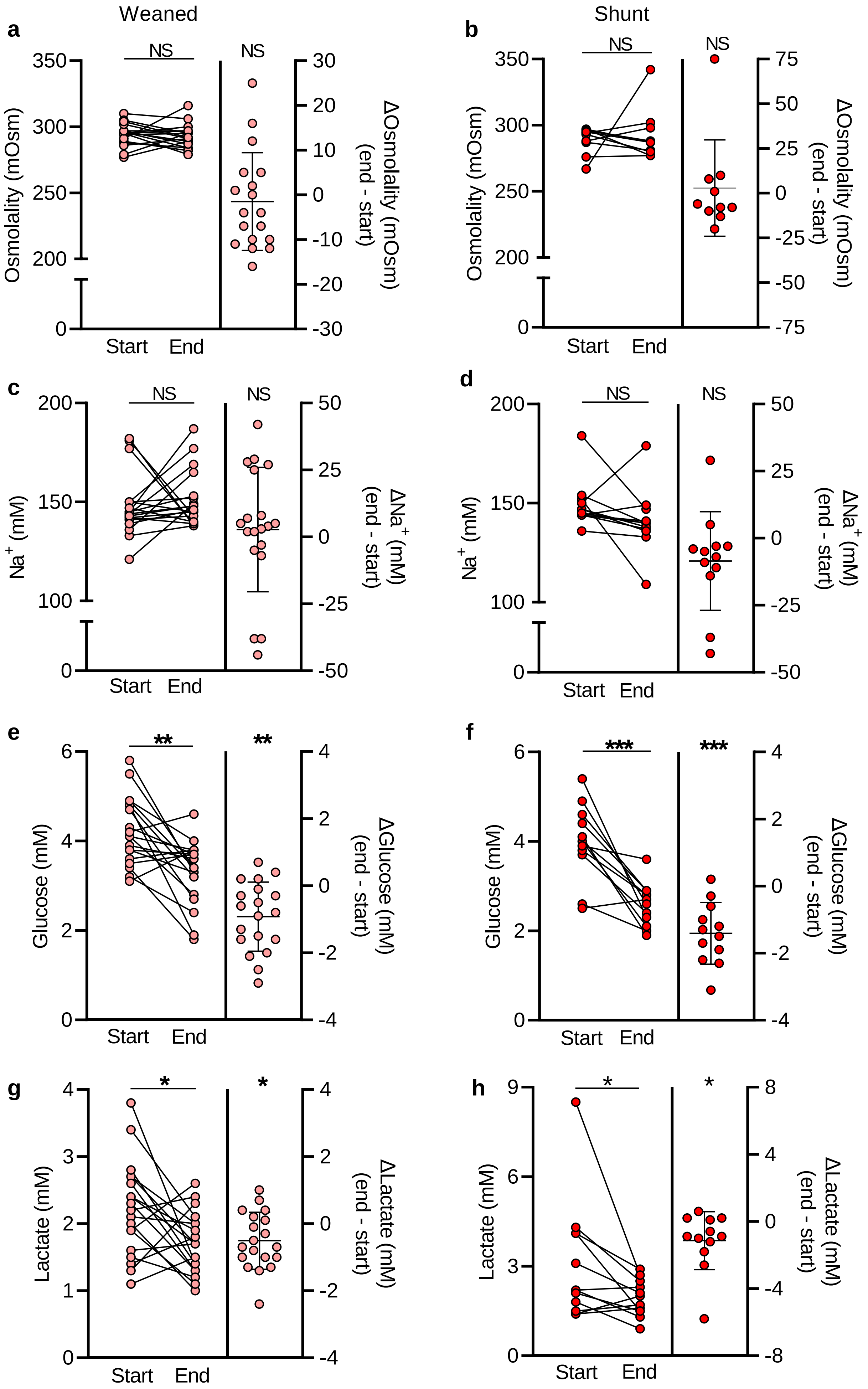

Supplementary Figure S3

Figure S3. CSF osmolality, Na^+, glucose, and lactate content in weaned and shunted SAH patients. (**a**) CSF osmolality (left) in start samples (294 ± 8 mOsm) and end samples (293 ± 9 mOsm) collected from weaned SAH patients (n = 18 of each, P = 0.57). Average CSF osmolality change is depicted on the right (2 ± 11 mOsm, n = 18, P = 0.57). (**b**) CSF osmolality (left) in start samples (289 ± 10 mOsm) and end samples (292 ± 20 mOsm) collected from shunted SAH patients (n = 10 of each, P = 0.58). Average CSF osmolality change is depicted on the right (3 ± 27 mOsm, n = 10, P = 0.58). (**c**) CSF Na^+ concentration (left) in start samples (148 ± 16 mM) and end samples (151 ± 14 mM) collected from weaned SAH patients (n = 19 of each, P = 0.37). Average CSF Na^+ change is depicted on the right (3 ± 23 mM, n = 19, P = 0.61). (**d**) CSF Na^+ concentration (left) in start samples (149 ± 12 mM) and end samples (141 ± 16 mM) collected from shunted SAH patients (n = 12 of each, P = 0.05). Average CSF Na^+ change is depicted on the right (9 ± 18 mM, n = 12, P = 0.14). (**e**) CSF glucose levels (left) in start samples (4.23 ± 0.76 mM) and end samples (3.31 ± 0.71 mM) collected from weaned SAH patients (n = 19 of each, P < 0.01). Average CSF glucose decrease is depicted on the right (0.92 ± 1.03 mM, n = 19, P < 0.01). (**f**) CSF glucose levels (left) in start samples (3.99 ± 0.83 mM) and end samples (2.58 ± 0.48 mM) collected from shunted SAH patients (n = 12 of each, P < 0.001). Average CSF glucose decrease is depicted on the right (1.41 ± 0.92 mM, n = 12, P < 0.001). (**g**) CSF lactate levels (left) in start samples (2.22 ± 0.70 mM) and end samples (1.71 ± 0.45 mM) collected from weaned SAH patients (n = 19 of each, P < 0.05). Average CSF lactate decrease is depicted on the right (0.51 ± 0.85 mM, n = 19, P < 0.05). (**h**) CSF lactate levels (left) in start samples (3.06 ± 2.03 mM) and end samples (1.92 ± 0.60 mM) collected from shunted SAH patients (n = 12 of each, P < 0.05). Average CSF lactate decrease is depicted on the right (1.14 ± 1.73 mM, n = 12, P < 0.05). Data are presented as mean ± standard deviation. For paired CSF samples, statistical significance was tested with a paired two-tailed t-test or a Wilcoxon matched-pairs signed rank test, depending on normality. For determination of average changes, a one-sample two-tailed t-test or a one-sample Wilcoxon signed rank test was conducted, depending on normality. * P < 0.05. ** P < 0.01. *** P < 0.001. NS = not significant.
